# Four decades of research on alexithymia: moving toward clinical applications

**DOI:** 10.3389/fpsyg.2013.00861

**Published:** 2013-11-19

**Authors:** Dalya Samur, Mattie Tops, Caroline Schlinkert, Markus Quirin, Pim Cuijpers, Sander L. Koole

**Affiliations:** ^1^Department of Clinical Psychology, VU University AmsterdamAmsterdam, Netherlands; ^2^Institute of Psychology, University of OsnabrückOsnabrück, Germany

**Keywords:** alexithymia, treatment, implications, oxytocin, neurofeedback, emotion processing

Virtually everyone has experienced from time to time how hard it can be to put one's feelings into words. Yet, for some individuals, this task is especially daunting. Such individuals are characterized by high levels of alexithymia (“no words for feelings”). *Alexithymia* is a personality dimension that involves both *cognitive deficits*, including difficulties in recognizing, describing, and distinguishing feelings from bodily sensations of emotional arousal, and *affective deficits*, including difficulties in emotionalizing and fantasizing (Bermond et al., 2007). Alexithymia is implicated in a wide variety of psychological problems, such as depression (Honkalampi et al., 2000) and schizophrenia (Rotenberg, 1994). Moreover, emotional deficits in autism spectrum disorder may be largely driven by alexithymia (Bird and Cook, 2013). Finally, alexithymia is associated with suicidality (Hintikka et al., 2004), increased psychosomatic complaints (Lane, 2008) and elevated mortality rates (Tolmunen et al., 2010).

Since the term “alexithymia” was introduced some four decades ago by Sifneos (1973), individual differences in alexithymia have been the focus of considerable research efforts. Indeed, at the time of writing this article (October 14, 2013), we counted more than 1400 articles in the PsychInfo database with “alexithymia” in the title or abstract. This research has analyzed many different facets of alexithymia, including its behavioral, linguistic, physiological, and neurological correlates (for recent reviews, see Taylor and Bagby, 2004; Kano and Fukudo, 2013; Nowakowski et al., 2013; van der Velde et al., 2013) as well as its assessment techniques (for a review, see Lumley et al., 2007). The accumulated knowledge based on the formation and assessment provides valuable insights into alexithymia. Nevertheless, researchers have so far taken few steps to translate these insights into treatments and interventions.

The current lack of translational work in alexithymia research seems highly unfortunate, considering that epidemiological studies indicate that as many as 10% of the general population may be characterized by levels of alexithymia that are sufficiently high to qualify as pathological (Salminen et al., 1999). Basic research remains necessary to answer unresolved questions about alexithymia. Nevertheless, a number of empirical findings on alexithymia now seem sufficiently well-established to consider their clinical application. Ogrodniczuk et al. (2011) found that high-alexithymic individuals seeking a treatment are as open as low-alexithymic individuals to receiving psychotherapy. Moreover, several studies have shown that alexithymia scores may decline during psychotherapy and such changes are correlated with improvement in therapy. For instance, high-alexithymic individuals may benefit from cognitive behavioral therapy (Spek et al., 2008) and also seem responsive to group therapy (e.g., Beresnevaite, 2000; Ogrodniczuk et al., 2011). As Ogrodniczuk et al., concluded, “This implies that alexithymic patients can at least partly develop some capacity to recognize their feelings and to communicate them to other people, thus enhancing their ability to use emotional information to guide adaptive behavior.”

Nevertheless, to the best of our knowledge, there exists no treatment specifically designed to overcome the problems associated with alexithymia. Furthermore, many existing forms of psychotherapy may be less than optimal for helping high-alexithymic individuals, given that they typically achieve poorer outcomes in psychotherapy than low-alexithymic individuals (Bach and Bach, 1995; McCallum et al., 2003; Ogrodniczuk et al., 2011). It therefore seems prudent to develop treatments that are specifically designed to overcome the problems associated with alexithymia. Offering such treatments to high-alexithymic individuals may significantly boost the effectiveness of psychotherapy within this group.

We therefore call upon researchers to consider more systematically how basic research findings may be translated into tools for improving the fate of alexithymic individuals. To facilitate the translation process, we briefly describe some promising ways in which alexithymia research may be converted into clinical interventions. Notably, our discussion is selective rather than exhaustive. We aim to identify the “low hanging fruit” that can be profitably explored by researchers who are interested in developing more effective evidence-based treatments for alexithymia.

## Alexithymia and emotion processing

Deficits in emotional processing are central to the notion of alexithymia. Indeed, behavioral experiments have shown that alexithymia is linked to poorer recognition of emotional expressions in faces (Grynberg et al., 2012; Cook et al., 2013) and lower recall for emotional material (Luminet et al., 2006). One straightforward clinical use of these tasks may lie in helping practitioners to refine their diagnosis of alexithymia. To this end, it will be important to develop standardized emotion processing tasks that have norm scores for appropriate populations.

Beyond diagnosis, however, an exciting possibility is that emotional processing tasks may be used to train emotional skills among alexithymic individuals. Here, an analogy may be drawn with research on attentional biases in individuals with anxiety disorders (see Mathews and MacLeod, 2002). Initial research showed that high-anxious individuals display an attentional bias toward threatening stimuli. The same tasks that were used to demonstrate this attentional bias were subsequently modified by researchers to train high-anxious individuals to shift their attention away from threatening stimuli. In an analogous manner, experimental tasks that have so far been used to demonstrate deficient emotion processes among alexithymic individuals may be modified to overcome these deficiencies. For instance, alexithymic individuals may be trained to better recognize emotional expressions in faces (see Cook et al., 2013, for a relevant task) and to have better recollection of emotional memories (see Luminet et al., 2006). Examining whether such training tasks may benefit alexithymic individuals seems a worthy question for future research.

## Alexithymia and language

Among human beings, the ability to express emotions requires processing at the linguistic level. Thus, linguistic processing plays a key role in alexithymia. Indeed, alexithymic people display impaired processing of emotional language at multiple levels. At a basic perceptual level, alexithymic people display poorer sensitivity to the emotional meanings of language. For instance, relative to low-alexithymic individuals, high-alexithymic individuals showed less facilitation from priming emotional contexts on the processing of emotion words (Suslow and Junghanns, 2002). Alexithymic people are further impaired in the perception and processing of speech prosody, or melody of speech, with emotional content (Goerlich et al., 2013). One possible linguistic intervention might consist of training alexithymic individuals in affect labeling that is, in lexicalizing emotional aspects of emotional stimuli and events. Research among general populations (which did not assess alexithymia) indicates that affect labeling improves emotion regulation (Lieberman et al., 2011). Thus, affect labeling could be an effective antidote against alexithymia.

At a communicative level, alexithymic individuals further demonstrate problems in emotional language production and comprehension. In particular, alexithymic individuals display a limited ability to talk about interpersonal relationships (Meganck et al., 2009), describe others' emotional experiences (Bydlowski et al., 2005), and understand the emotions of others (Moriguchi et al., 2006; Swart et al., 2009). In personal narratives, alexithymic individuals tend to use vocabulary of limited complexity and their emotional discourse lacks any vivid descriptions (Meganck et al., 2009). Alexithymia is also linked to concretist thinking and avoidance of metaphors (Kreitler, 2002).

The impoverished linguistic style of alexithymic individuals may be enriched by training them to use metaphors (Kousta et al., 2011) and mental imagery (Holmes and Mathews, 2010). A recent series of innovative experiments by Kidd and Castano (2013) suggests that reading literary fiction (as opposed to non-literary texts) can improve people's ability to identify and understand other's subjective states. These are among the primary social-cognitive abilities that are impaired among alexithymic individuals. As such, the intriguing possibility arises that alexithymic individuals may overcome some of their communicative difficulties by reading works of literary fiction, and perhaps also by receiving appropriate training in literary skills (e.g., poetry or creative writing).

## Oxytocin and neurofeedback

Recent years have seen a rapid growth of physiological and neuroscientific studies of alexithymia. Even though research within these areas is relatively novel, some of this research shows some promising therapeutic potential.

The neuropeptide oxytocin has been found to alter the perceptual salience and/or processing of social cues and to increase socio-emotional communication and trust (Carter, 1998; Bartz et al., 2011). Although oxytocin is naturally produced by the body, it can also be externally administered by inhaling it through the nose. Administration studies indicate that oxytocin fosters social-cognitive processes that are impaired among alexithymic individuals. For instance, a recent study showed that intranasal oxytocin increases people's willingness to verbally share painful emotions (Lane et al., 2013). Importantly, oxytocin did not simply make people more talkative but instead specifically increased the willingness to share emotions. Preclinical and clinical studies have identified important links between oxytocin and a range of psychiatric disorders, and have now started to directly assess its therapeutic potential in treating socio-emotional functioning deficits (Matsuzaki et al., 2012; Macdonald and Feifel, 2013; Tops et al., 2013). Most relevant here, a recent study suggests that the social-cognitive benefits of oxytocin are particularly pronounced among individuals who score high (rather than low) on alexithymia (Luminet et al., 2011). Oxytocin may impact on central aspects of alexithymia such as the decreased recognition, expression, and consequently sharing of emotions. Although more research is clearly needed, these preliminary findings suggest that intranasal oxytocin might have therapeutic benefits, perhaps as an adjunct to therapy or training.

Neuroimaging research has further linked alexithymia to decreased neural activations to emotional stimuli [see van der Velde et al. (2013), for a meta-analysis]. Interventions might target these specific neural areas using specialized techniques. For instance, online transcranial magnetic stimulation can be used to activate brain areas that are involved in emotion as well as language [e.g., the inferior frontal gyrus (IFG); Hartwigsen et al., 2010; Hoekert et al., 2010]. Moreover, brain areas (e.g., the anterior insula) that have been implicated in empathy, emotion, interoceptive awareness, and alexithymia (e.g., Bird et al., 2010; Bernhardt et al., 2013) and areas involved in emotion and language (IFG; Rota et al., 2009) can be trained by neurofeedback using real-time functional magnetic resonance imaging (e.g., Caria et al., 2010). Alternatively, neurofeedback may be provided through low-cost technology such as near-infrared spectroscopy (Mihara et al., 2012), which can detect relevant functional activation in areas such as the IFG (Takei et al., 2013). Training of anterior insula activation also changes the appraisal of emotional stimuli (Caria et al., 2010) and in patients with schizophrenia it led to changes in the perception of emotions and modulations of the brain network connectivity (Ruiz et al., 2013). These findings open the door to further studies in alexithymia and psychiatric populations, and possible therapeutic applications.

## Conclusions and outlook

The pains of putting one's feelings into words are a universal human experience that lies at heart of the personality dimension of alexithymia. Over the last four decades, the alexithymia construct has inspired volumes of research. The resulting wealth of empirical findings has illuminated many aspects of alexithymia, but so far has not resulted in the development of new, evidence-based treatments for improving the life of alexithymic individuals. Nevertheless, as we have outlined in this article, behavioral, linguistic, and neuroscience research on alexithymia seem to have progressed to a point where they may be translated into effective treatments for alexithymic individuals. These treatments may be delivered in innovative formats, such as Internet-based programs. These programs might be especially appealing to alexithymic individuals, because online communication provides a way to keep interpersonal contact at a minimum, lowering the need for openly sharing one's emotions. Treatments for alexithymia may be offered to complement existing clinical treatments, to permit high alexithymic individuals to derive more benefits from psychotherapy.

With this outline, we hope to stimulate researchers to invest more in the development of evidence-based treatments for alexithymia, and to evaluate these treatments in terms of their effectiveness. The findings of evaluative research may in turn inform basic research, creating a dynamic dialogue between practitioners and basic researchers. After four decades of basic research on alexithymia, the time seems ripe to move toward clinical applications.

## References

[B1] Bach M. Bach D. (1995). Predictive value of alexithymia: a prospective study in somatizing patients. Psychother. Psychosom. 64, 43–48 10.1159/000288989 7480582

[B2] Bartz J. A. Zaki J. Bolger N. Ochsner K. N. (2011). Social effects of oxytocin in humans: context and person matter. Trends. Cogn. Sci. 15, 301–309 10.1016/j.tics.2011.05.002 21696997

[B3] Beresnevaite M. (2000). Exploring the benefits of group psychotherapy in reducing alexithymia in coronary heart disease patients: a preliminary study. Psychother. Psychosom. 69, 117–122 10.1159/000012378 10773774

[B4] Bermond B. Clayton K. Liberova A. Luminet O. Maruszewski T. Ricci Bitti P. E. (2007). A cognitive and an affective dimension of alexithymia in six languages and seven populations. Cogn. Emot. 21, 1125–1136 10.1080/02699930601056989

[B5] Bernhardt B. C. Valk S. L. Silani G. Bird G. Frith U. Singer T. (2013). Selective disruption of sociocognitive structural brain networks in autism and alexithymia. Cereb. Cortex. [Epub ahead of print]. 10.1093/cercor/bht182 23863687

[B6] Bird G. Cook R. (2013). Mixed emotions: the contribution of alexithymia to the emotional symptoms of autism. Transl. Psychiatry 3, e285 10.1038/tp.2013.61 23880881 PMC3731793

[B7] Bird G. Silani G. Brindley R. White S. Frith U. Singer T. (2010). Empathic brain responses in insula are modulated by levels of alexithymia but not autism. Brain 133, 1515–1525 10.1093/brain/awq060 20371509 PMC2859151

[B8] Bydlowski S. Corcos M. Jeammet P. Paterniti S. Berthoz S. Laurier C. (2005). Emotion-processing deficits in eating disorders. Int. J. Eat. Disord. 37, 321–329 10.1002/eat.20132 15856501

[B9] Caria A. Sitaram R. Veit R. Begliomini C. Birbaumer N. (2010). Volitional control of anterior insula activity modulates the response to aversive stimuli. A real-time functional magnetic resonance imaging study. Biol. Psychiatry 68, 425–432 10.1016/j.biopsych.2010.04.020 20570245

[B10] Carter C. S. (1998). Neuroendocrine perspectives on social attachment and love. Psychoneuroendocrinology 23, 779–818 10.1016/S0306-4530(98)00055-9 9924738

[B11] Cook R. Brewer R. Shah P. Bird G. (2013). Alexithymia, not autism, predicts poor recognition of emotional facial expressions. Psychol. sci. 24, 723–732 10.1177/0956797612463582 23528789

[B12] Goerlich K. S. Witteman J. Schiller N. O. van Heuven V. J. Aleman A. Martens S. (2013). Blunted feelings: alexithymia is associated with a diminished neural response to speech prosody. Soc. Cogn. Affect. Neurosci. [Epub ahead of print]. 10.1093/scan/nst075 23681887 PMC4127007

[B13] Grynberg D. Chang B. Corneille O. Maurage P. Vermeulen N. Berthoz S. (2012). Alexithymia and the processing of emotional facial expressions (EFEs): systematic review, unanswered questions and further perspectives. PLoS ONE 7:e42429 10.1371/journal.pone.0042429 22927931 PMC3426527

[B14] Hartwigsen G. Price C. J. Baumgaertner A. Geiss G. Koehnke M. Ulmer S. (2010). The right posterior inferior frontal gyrus contributes to phonological word decisions in the healthy brain: evidence from dual-site TMS. Neuropsychologia 48, 3155–3163 10.1016/j.neuropsychologia.2010.06.032 20600177 PMC3223523

[B15] Hintikka J. Honkalampi K. Koivumaa-Honkanen H. Antikainen R. Tanskanen A. Haatainen K. (2004). Alexithymia and suicidal ideation: a 12-month follow-up study in a general population. Compr. Psychiatry 45, 340–345 10.1016/j.comppsych.2004.06.008 15332196

[B16] Hoekert M. Vingerhoets G. Aleman A. (2010). Results of a pilot study on the involvement of bilateral inferior frontal gyri in emotional prosody perception: an rTMS study. BMC Neurosci. 11:93 10.1186/1471-2202-11-93 20698964 PMC2925361

[B17] Holmes E. A. Mathews A. (2010). Mental imagery in emotion and emotional disorders. Clin. Psychol. Rev. 30, 349–362 10.1016/j.cpr.2010.01.001 20116915

[B18] Honkalampi K. Hintikka J. Tanskanen A. Lehtonen J. Viinamäki H. (2000). Depression is strongly associated with alexithymia in the general population. J. Psychosom. Res. 48, 99–104 10.1016/S0022-3999(99)00083-5 10750635

[B19] Kano M. Fukudo S. (2013). The alexithymic brain: the neural pathways linking alexithymia to physical disorders. Biopsychosoc. Med. 7:1 10.1186/1751-0759-7-1 23302233 PMC3563604

[B20] Kidd D. C. Castano E. (2013). Reading literary fiction improves theory of mind. Science 342, 377–380 10.1126/science.1239918 24091705

[B21] Kousta S. T. Vigliocco G. Vinson D. P. Andrews M. Del Campo E. (2011). The representation of abstract words: why emotion matters. J. Exp. Psychol. Gen. 140, 14 10.1037/a0021446 21171803

[B22] Kreitler S. (2002). The psychosemantic approach to alexithymia. Pers. Individ. Diff. 33, 393–407 10.1016/S0191-8869(01)00163-5

[B23] Lane A. Luminet O. Rimé B. Gross J. J. de Timary P. Mikolajczak M. (2013). Oxytocin increases willingness to socially share one's emotions. Int. J. Psychol. 48, 676–681 10.1080/00207594.2012.677540 22554106

[B24] Lane R. D. (2008). Neural substrates of implicit and explicit emotional processes: a unifying framework for psychosomatic medicine. Psychosom. Med. 70, 214–231 10.1097/PSY.0b013e3181647e44 18256335

[B25] Lieberman M. D. Inagaki T. K. Tabibnia G. Crockett M. J. (2011). Subjective responses to emotional stimuli during labeling, reappraisal, and distraction. Emotion 11, 468 10.1037/a0023503 21534661 PMC3444304

[B26] Luminet O. Grynberg D. Ruzette N. Mikolajczak M. (2011). Personality-dependent effects of oxytocin: greater social benefits for high alexithymia scorers. Biol. Psychol. 87, 401–406 10.1016/j.biopsycho.2011.05.005 21641964

[B27] Luminet O. Vermeulen N. Demaret C. Taylor G. J. Bagby R. M. (2006). Alexithymia and levels of processing: evidence for an overall deficit in remembering emotion words. J. Res. Pers. 40, 713–733 10.1016/j.jrp.2005.09.001

[B28] Lumley M. A. Neely L. C. Burger A. J. (2007). The assessment of alexithymia in medical settings: implications for understanding and treating health problems. J. Pers. Assess. 89, 230–246 10.1080/00223890701629698 18001224 PMC2931418

[B29] Macdonald K. Feifel D. (2013). Helping oxytocin deliver: considerations in the development of oxytocin-based therapeutics for brain disorders. Front. Neurosci. 7:35 10.3389/fnins.2013.00035 23508240 PMC3597931

[B30] Mathews A. MacLeod C. (2002). Induced processing biases have causal effects on anxiety. Cogn. Emot. 16, 331–354 10.1080/0269993014300051815325899

[B31] Matsuzaki M. Matsushita H. Tomizawa K. Matsui H. (2012). Oxytocin: a therapeutic target for mental disorders. J. Physiol. Sci. 62, 441–444 10.1007/s12576-012-0232-9 23007624 PMC10717158

[B32] McCallum M. Piper W. E. Ogrodniczuk J. S. Joyce A. S. (2003). Relationships among psychological mindedness, alexithymia and outcome in four forms of short term psychotherapy. Psychol. Psychother. 76, 133–144 10.1348/147608303765951177 12855060

[B33] Meganck R. Vanheule S. Inslegers R. Desmet M. (2009). Alexithymia and interpersonal problems: a study of natural language use. Pers. Individ. Diff. 47, 990–995 10.1016/j.paid.2009.08.005

[B34] Mihara M. Miyai I. Hattori N. Hatakenaka M. Yagura H. Kawano T. (2012). Neurofeedback using real-time near-infrared spectroscopy enhances motor imagery related cortical activation. PLos ONE 7:e32234 10.1371/journal.pone.0032234 22396753 PMC3292558

[B35] Moriguchi Y. Ohnishi T. Lane R. D. Maeda M. Mori T. Nemoto K. (2006). Impaired self-awareness and theory of mind: an fMRI study of mentalizing in alexithymia. Neuroimage 32, 1472–1482 10.1016/j.neuroimage.2006.04.186 16798016

[B36] Nowakowski M. E. McFarlane T. Cassin S. (2013). Alexithymia and eating disorders: a critical review of the literature. J. Eat. Disord. 1, 21 10.1186/2050-2974-1-21 24999402 PMC4081716

[B37] Ogrodniczuk J. S. Piper W. E. Joyce A. S. (2011). Effect of alexithymia on the process and outcome of psychotherapy: a programmatic review. Psychiatry Res. 190, 43–48 10.1016/j.psychres.2010.04.026 20471096

[B38] Rota G. Sitaram R. Veit R. Erb M. Weiskopf N. Dogil G. (2009). Self-regulation of regional cortical activity using real-time fMRI: the right inferior frontal gyrus and linguistic processing. Hum. Brain Mapp. 30, 1605–1614 10.1002/hbm.20621 18661503 PMC6870622

[B39] Rotenberg V. S. (1994). An integrative psychophysiological approach to brain hemisphere functions in schizophrenia. Neurosci. Biobehav. Rev. 18, 487–495 10.1016/0149-7634(94)90003-5 7708362

[B40] Ruiz S. Lee S. Soekadar S. R. Caria A. Veit R. Kircher T. (2013). Acquired self-control of insula cortex modulates emotion recognition and brain network connectivity in schizophrenia. Hum. Brain Mapp. 34, 200–212 10.1002/hbm.21427 22021045 PMC6869886

[B41] Salminen J. K. Saarijärvi S. Äärelä E. Toikka T. Kauhanen J. (1999). Prevalence of alexithymia and its association with sociodemographic variables in the general population of Finland. J. Psychosom. Res. 1, 75–82 10.1016/S0022-3999(98)00053-1 10088984

[B42] Sifneos P. E. (1973). The prevalence of ‘alexithymic’ characteristics in psychosomatic patients. Psychother. Psychosom. 22, 255–262 10.1159/000286529 4770536

[B43] Spek V. Nyklíèek I. Cuijpers P. Pop V. (2008). Alexithymia and cognitive behaviour therapy outcome for subthreshold depression. Acta Psychiatr. Scand. 118, 164–167 10.1111/j.1600-0447.2008.01199.x 18498434

[B44] Suslow T. Junghanns K. (2002). Impairments of emotion situation priming in alexithymia. Pers. Individ. Diff. 32, 541–550 10.1016/S0191-8869(01)00056-3

[B45] Swart M. Kortekaas R. Aleman A. (2009). Dealing with feelings: characterization of trait alexithymia on emotion regulation strategies and cognitive-emotional processing. PLoS ONE 4:e5751 10.1371/journal.pone.0005751 19492045 PMC2685011

[B46] Takei Y. Suda M. Aoyama Y. Yamaguchi M. Sakurai N. Narita K. (2013). Temporal lobe and inferior frontal gyrus dysfunction in patients with schizophrenia during face-to-face conversation: a near-infrared spectroscopy study. J. Psychiatr. Res. 47, 1581–1589 10.1016/j.jpsychires.2013.07.029 23978395

[B47] Taylor G. J. Bagby R. M. (2004). New trends in alexithymia research. Psychother. Psychosom. 73, 68–77 10.1159/000075537 14767148

[B48] Tolmunen T. Lehto S. M. Heliste M. Kurl S. Kauhanen J. (2010). Alexithymia is associated with increased cardiovascular mortality in middle-aged Finnish men. Psychosom. Med. 72, 187–191 10.1097/PSY.0b013e3181c65d00 19949161

[B49] Tops M. Koole S. L. IJzerman H. Buisman-Pijlman F. T. A. (2013). Why social attachment and oxytocin protect against addiction and stress: insights from the dynamics between ventral and dorsal corticostriatal systems. Pharmacol. Biochem. Behav. 4:761 10.1016/j.pbb.2013.07.015 23916423

[B50] van der Velde J. Servaas M. N. Goerlich K. S. Bruggeman R. Horton P. Costafreda S. G. (2013). Neural correlates of alexithymia: a meta-analysis of emotion processing studies. Neurosci. Biobehav. Rev. 37, 1774–1785 10.1016/j.neubiorev.2013.07.008 23886515

